# Renal Outcomes After Sleeve Gastrectomy versus Gastric Bypass in Patients with Nephropathy: Results from a Retrospective Study

**DOI:** 10.1007/s11695-026-08684-4

**Published:** 2026-04-21

**Authors:** Emilia Lutz, Adisa Poljo, Ulrike Heger, Beat P Müller, Adrian T Billeter

**Affiliations:** 1 Department of Anesthesiology, University Hospital Heidelberg, Heidelberg, Germany; 2 Department of General, Visceral and Transplantation Surgery, University Hospital Heidelberg, Heidelberg, Germany; 3 Department of Visceral Surgery, University Hospital of Basel, Basel, Switzerland; 4 Department of Visceral Surgery, Clarunis, University Digestive Health Care Center Basel, St. Clara Hospital and University Hospital Basel, Basel, Switzerland

**Keywords:** Metabolic bariatric surgery, Sleeve gastrectomy, Roux-en-Y gastric bypass, Chronic kidney disease, Albuminuria, Serum uromodulin

## Abstract

**Introduction:**

The influence of metabolic bariatric surgery (MBS) on renal damage remains poorly understood. Data on the impact of different types of MBS on renal markers is limited. This study analyzes the effects of sleeve gastrectomy (SG) and Roux-en-Y gastric bypass (RYGB) on traditional (urinary albumin-creatinine ratio (uACR), estimated glomerular filtration rate (eGFR)) and new markers (serum uromodulin (sUMOD), serum kidney injury molecule-1 (sKIM-1)) of kidney function in a retrospective cohort of patients with nephropathy.

**Methods:**

50 patients (RYGB *n* = 25; SG *n* = 25) with renal involvement, defined as microalbuminuria (uACR > 3 mg/mmol) and/or reduced eGFR (< 60 ml/min) were included. Patients were matched for sex, age, body mass index (BMI) and glycemic control. Kidney function was assessed using creatinine, cystatin C, eGFR, uACR, sUMOD and sKIM-1.

**Results:**

After a mean follow-up of 28.8 ± 6.1 months, renal involvement completely resolved in 48% after SG (*p*<.001) compared to 68% after RYGB (*p*<.001). UACR decreased after both procedures (SG -9.9 ± 23.2 mg/mmol, *p*=.002; RYGB − 14.4 ± 28.9 mg/mmol, *p*<.001) without a difference between procedures (*p*=.923). SUMOD and sKIM-1 improved significantly after SG (*p*=.025, *p*=.028).

**Conclusions:**

In this study, MBS was associated with improved nephropathy outcomes, independent of the type of procedure performed. SUMOD emerges as a promising biomarker to predict renal recovery.

**Supplementary Information:**

The online version contains supplementary material available at https://doi.org/10.1007/s11695-026-08684-4.

## Key Points

SG and RYGB both improve nephropathy, with similar effectiveness.

Patients without arterial hypertension and type 2 diabetes mellitus show the greatest rate of kidney recovery, accompanied by significant increases in serum uromodulin.

Serum uromodulin is a promising biomarker that predicts renal recovery better than traditional kidney function markers after metabolic bariatric surgery.

**Supplementary Information:**

The online version contains supplementary material available at https://doi.org/10.1007/s11695-026-08684-4.

## Introduction

Obesity is an independent risk factor for chronic kidney disease (CKD). Obesity-related renal damage is linked to glomerular hypertrophy and sclerosis, inflammation and additional damage related to type 2 diabetes mellitus (T2D) and arterial hypertension [1–3]. The global surge in obesity and the concomitant rise in CKD cause a considerable burden for healthcare worldwide [3–5]. Metabolic bariatric surgery (MBS), predominantly sleeve gastrectomy (SG) and Roux-en-Y gastric bypass (RYGB), effectively addresses CKD risk factors by reducing weight, blood pressure, insulin resistance and proteinuria [6]. To date, no metabolic procedure has demonstrated a clear advantage in improving or preventing end-organ damage [7]. While there is evidence suggesting that MBS may have a renoprotective effect in CKD [8], the specific impacts of different procedures on nephropathy have yet to be thoroughly investigated. When considering T2D, MBS is recognized as a more effective treatment for complications compared to medical therapy [9, 10]. Additionally, some studies indicate that MBS may have a positive impact on renal function in patients with obesity and T2D [11, 12]. In a randomized trial, RYGB resulted in a greater reduction in albuminuria at 2 years compared to medical therapy. However, this advantage diminished by the 5-year follow-up [13, 14]. In contrast, other metabolic procedures, such as SG, and non-diabetic causes of nephropathy have not been adequately investigated. As such, further research is warranted to compare SG and RYGB in patients with nephropathy and to evaluate the effects of MBS across various etiologies of kidney disease. Common surrogate markers of kidney function, like creatinine, cystatin C, and estimated glomerular filtration rate (eGFR), have important limitations in assessing renal function, especially in patients who experience major ophysiological changes following MBS [15]. Therefore, there is a need for alternative markers that provide direct assessment of kidney function. Serum uromodulin (sUMOD) has been identified as a sensitive kidney-specific biomarker that reflects nephron mass and overall renal function [16]. Although evidence on sUMOD in the context of MBS is limited, initial findings are promising [17]. Another marker, kidney injury molecule-1 (KIM-1), a glycoprotein associated with renal damage, has been shown to improve after MBS, with changes correlating with improvement in albuminuria and serum creatinine levels [18, 19].

This study aims to evaluate the effects of SG versus RYGB on renal outcomes in patients with nephropathy and obesity. It uses both traditional and novel markers of kidney function, including serum creatinine, cystatin C, eGFR, urinary albumin-creatinine ratio (uACR), sUMOD, and sKIM-1. In addition to diabetic nephropathy, the study also investigates other subtypes of nephropathy.

## Materials and Methods

The study population was retrospectively selected from a prospectively maintained biobank of patients with obesity undergoing MBS at a specialized university hospital from 2017 to 2021. Inclusion criteria consisted of preoperative nephropathy, operationally defined as evidence of renal involvement indicated by uACR > 3 mg/mmol and/or eGFR < 60 ml/min/1.73 m² at baseline [20], and MBS procedures of SG and RYGB. Patients were matched for sex, age, body mass index (BMI) and glycated hemoglobin (HbA1c) in a nearest-neighbor propensity score matching in a 1:1 ratio. Cases of procedures other than SG and RYGB, metabolic conditions other than T2D, primary hormonal disorders, or those undergoing hemodialysis were excluded. Renal function was assessed using eGFR calculated by the Chronic Kidney Disease Epidemiology Collaboration combined creatinine-cystatin C equation (CKD-EPI formula), which has demonstrated accuracy in populations with obesity [21]. Detailed information on perioperative, surgical and laboratory processes is available in *Supplementary Materials*.

### Outcomes

The primary outcome was improvement of renal involvement, defined as normalization of eGFR (≥ 60 ml/min/1.73 m²) and/or remission of microalbuminuria, defined as a reduction of uACR to ≤ 3 mg/mmol. Secondary outcomes included changes in individual renal function markers – serum creatinine, cystatin C, eGFR, and uACR – as well as novel biomarkers serum uromodulin (sUMOD) and serum kidney injury molecule-1 (sKIM-1). Additional secondary outcomes included changes in glycemic control (HbA1c), blood pressure, use of antidiabetic and antihypertensive medication, body mass index (BMI) and total body weight loss (%TWL). Subgroup analyses were performed according to nephropathy subtype, including diabetic and non-diabetic forms.

### Statistical Analysis

Statistical analysis was conducted using IBM SPSS Statistics 28.0. (IBM Corp. Released 2021. IBM SPSS Statistics for Windows, Version 28.0. Armonk, NY: IBM Corp). Matching for propensity scores was performed using nearest-neighbor 1:1 matching without replacement to balance baseline characteristics between groups. Covariates included in the propensity score model were sex, age, preoperative BMI and HbA1c. The quality of the match was assessed by standardized mean differences (SMD), with |SMD| < 0.1 considered acceptable. Analyses were conducted on the matched pairs. Paired T-Test, McNemar test and Wilcoxon signed rank test were performed to compare pre- and postoperative values within a group and Mann-Whitney U, Kruskal-Wallis, Fisher and Chi-Square tests were used to compare different groups. Logistic regression analysis was applied for exploratory assessment of potential predictors. Given the limited sample size, results were interpreted as hypothesis-generating. Spearman correlation assessed linear correlation of ranked features. A significance level of 5% (α = 0.05, 2-sided) was applied in all statistical tests. Data are presented as mean ± standard deviation. Laboratory and clinical parameters are demonstrated in tables, bar charts and box blots. Graphs were created using Prism 5 (GraphPad Software, Inc., San Diego, CA, USA).

The study complies with the Declaration of Helsinki and received ethics committee approval. Written consent was obtained from all participants.

## Results

### Comparison of Sleeve Gastrectomy versus Roux-en-y Gastric Bypass

**Fig. 1:**
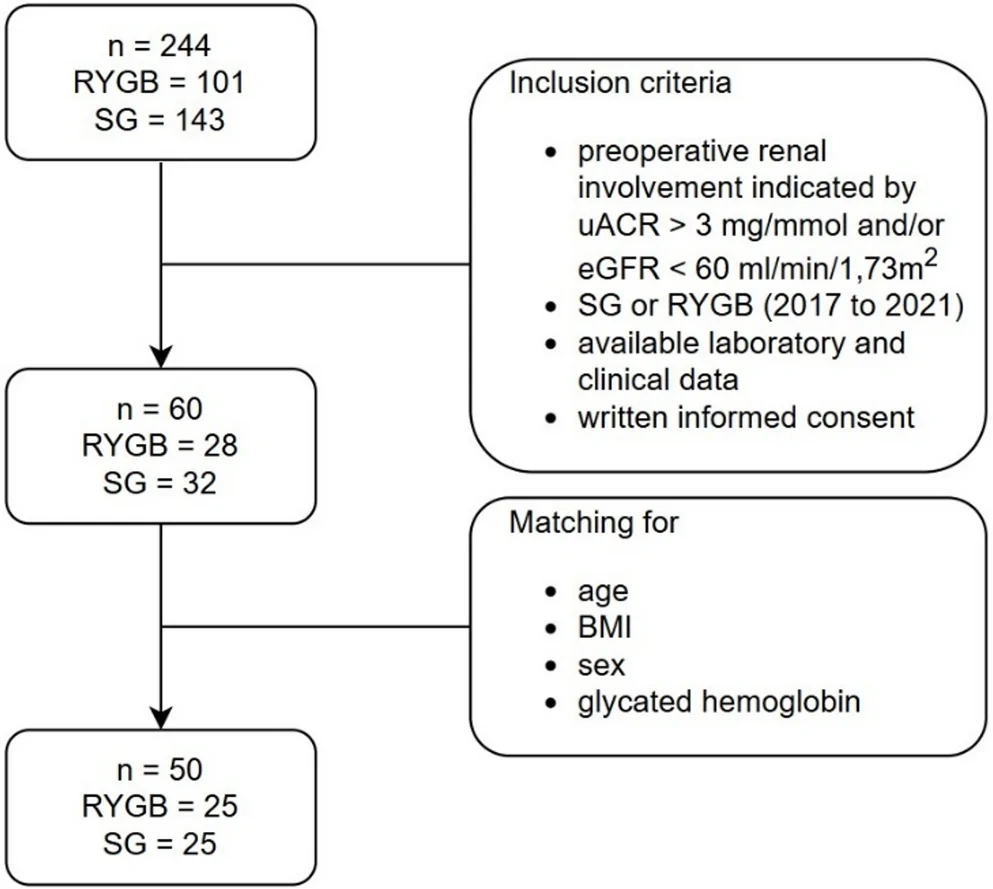
Retrospective patient selection process from prospectively maintained biobank. UACR = urinary albumin-creatinine ratio, eGFR = estimated glomerular filtration rate, SG = sleeve gastrectomy, RYGB = Roux-en-y gastric bypass, BMI = body mass index

**Fig. 2:**
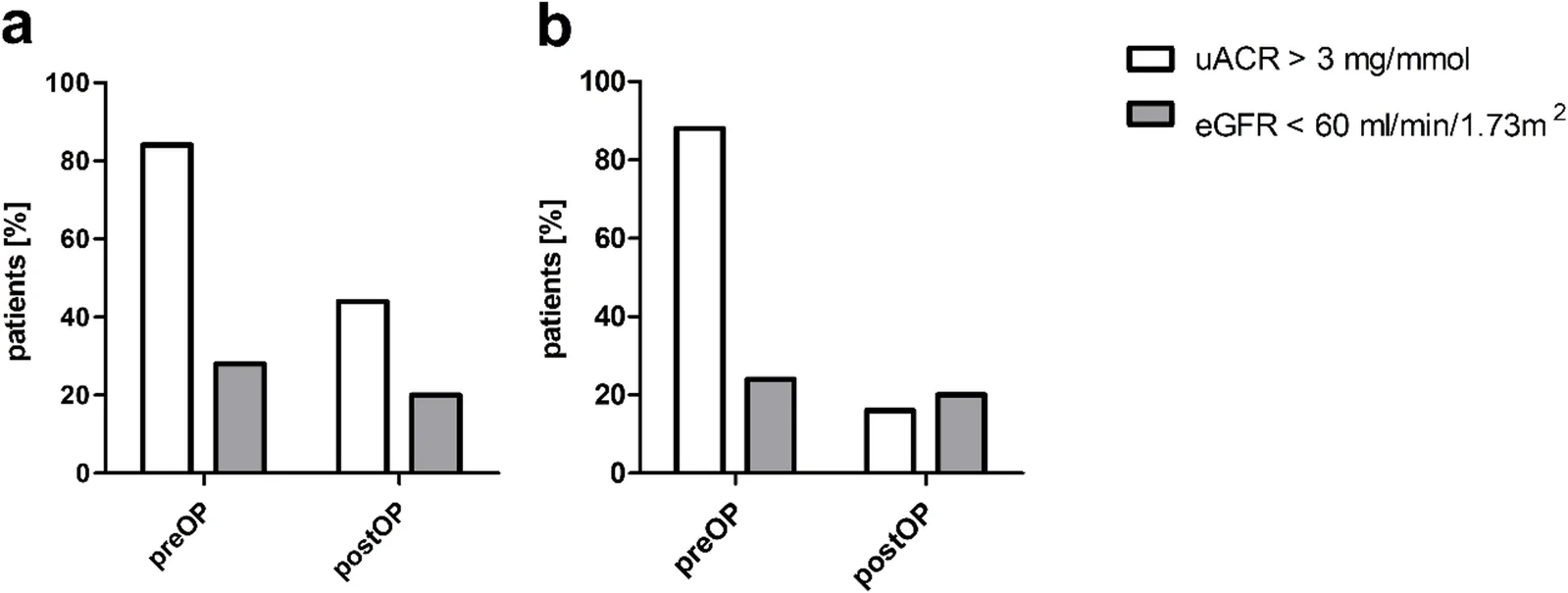
Pre- versus postoperative development of microalbuminuria (urinary albumin-creatinine ratio > 3 mg/mmol) and reduced eGFR (estimated glomerular filtration rate < 60 ml/min/1.73m^2^) in (**a**) SG (sleeve gastrectomy) and (**b**) RYGB (Roux-en-y gastric bypass) cohorts. White bars indicate albuminuria and shaded bars indicate reduced eGFR as defined above

**Fig. 3:**
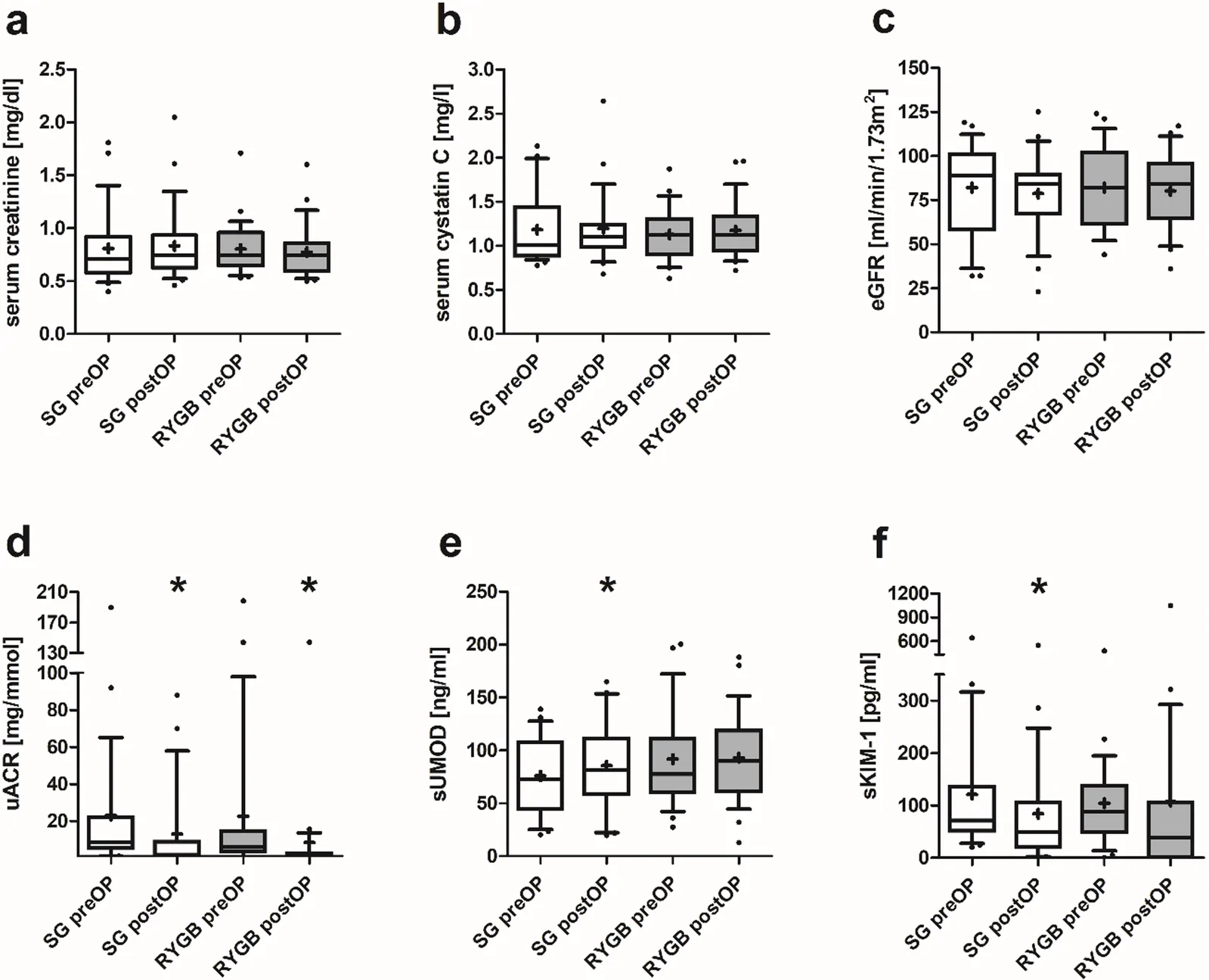
Development of (**a**) serum creatinine, (**b**) serum cystatin C, (**c**) estimated glomerular filtration rate (CKD-EPI combined creatinine-cystatin C formula), (**d**) urinary albumin-creatinine ratio, (**e**) serum uromodulin and (**f**) serum kidney injury molecule-1 in SG (sleeve gastrectomy, *n* = 25) and RYGB (Roux-en-y gastric bypass, *n* = 25) cohorts. * indicates *p* < .05. + indicates mean. Box plot whiskers mark 10 and 90 percentiles

A total of 244 patients were screened for preoperative nephropathy. Among these, 60 patients met the inclusion criteria and were included in the study. Patient selection is presented in the study flow chart in Fig. 1. After matching for sex, age, BMI and HbA1c, 25 cases each of SG and RYGB were included and underwent follow-up examination at approximately 2.5 years. Table 1 outlines baseline characteristics of both cohorts. There were no significant differences at baseline between SG and RYGB groups indicating effective matching. The mean follow-up was 28.8 ± 6.1 months. Resolution of renal involvement was achieved in 48% of patients undergoing SG (*p*<.001) and 68% of those undergoing RYGB (*p*<.001), with no statistically significant difference between the two procedures (*p* = .152). The rate of microalbuminuria remission (SG 47.6% vs. RYGB 81.8%) exceeded that of eGFR recovery (SG 28.6% vs. RYGB 16.7%), as shown in Fig. 2. UACR decreased significantly after SG (22.9 ± 39.8 to 13.0 ± 23.5 mg/mmol, *p* = .002) and RYGB (22.6 ± 47.3 to 8.3 ± 28.5 mg/mmol, *p* < .001). Similarly, both HbA1c (SG 6.9 ± 1.6 to 5.9 ± 1.1%, *p* < .001 vs. RYGB 7.3 ± 2.6 to 5.8 ± 1.3%, *p* < .001) and systolic blood pressure (SG 147.8 ± 16.7 to 138.4 ± 14.4 mmHg, *p* = .021 vs. RYGB 148.6 ± 20.3 to 132.9 ± 19.2 mmHg, *p* < .001) demonstrated significant reductions following both procedures. Although not primary renal endpoints, improvements in HbA1c and BMI reflect key drivers of kidney disease and support the biological plausibility of the observed renal changes. Reductions in UACR, HbA1c and systolic blood pressure were comparable between the two groups (*p* = .923; *p* = .346; *p* = .425, respectively). Patients undergoing RYGB experienced significantly greater %TWL than those undergoing SG (31.0 ± 11.6% vs. 23.6 ± 9.4%, *p* = .032). However, significant improvement in sUMOD and sKIM-1 were observed exclusively following SG (sUMOD 76.02 ± 35.80 to 85.56 ± 40.74 ng/ml, *p* = .025; sKIM-1 121.05 ± 134.69 to 84.40 ± 120.68 pg/ml, *p* = .028). All other evaluated parameters remained unchanged. Figure 3 visualizes the changes in kidney function parameters. Pre- and postoperative levels of all investigated parameters are presented in Table 2. Regarding medication, 8 out of 13 patients (61.5%) in the SG group who were on oral antidiabetics preoperatively were able to discontinue them postoperatively, while 4 (30.8%) required dose reductions. In the RYGB group, 12 out of 15 patients (80.0%) discontinued oral antidiabetics, and 2 patient (13.3%) had dose reductions. Insulin therapy was discontinued in 2 out of 5 cases (40.0%) cases following both procedures. For antihypertensive medications, 5 out of 18 patients (27.8%) in the SG group discontinued use, and 6 (33.3%) had dose reductions. After RYGB, antihypertensives were discontinued in 4 out of 14 patients (28.6%) and reduced in 8 (57.1%) cases.

**Table 1:** Baseline characteristics of included patients (SG vs. RYGB)

	SG (*n* = 25)	RYGB (*n* = 25)	*p*-value
Age, mean ± SD (yr)	48.0 ± 9.5	48.0 ± 8.9	*p* = .930
Sex, female/male (%)	72.0/28.0	72.0/28.0	*p* = 1.000
BMI, mean ± SD (kg/m^2^)	47.2 ± 5.3	47.1 ± 6.8	*p* = .946
HbA1c, mean ± SD (%)	6.9 ± 1.6	7.3 ± 2.6	*p* = .801
Patients with type 2 diabetes (%)	52.0	56.0	*p* = .777
Patients with impaired glucose tolerance or fasting glucose (%)	24.0	20.0	*p* = .733
Patients with antidiabetics (%)	52.0	60.0	*p* = .569
Patients with insulin therapy (%)	20.0	20.0	*p* = 1.000
Patients with hypertension (%)	80.0	68.0	*p* = .333
Patients with antihypertensives (%)	72.0	56.0	*p* = .239

*SG* Sleeve gastrectomy, *RYGB* Roux-en-y gastric bypass, *BMI *Body mass index, *HbA1C* Glycated hemoglobin.

**Table 2:** Pre- and postoperative levels of serum creatinine, cystatin C, eGFR, uACR, sUMOD, sKIM-1, HbA1c, blood pressure and BMI (SG vs. RYGB)

	SG	*p*-value	RYGB	*p*-value
Serum creatinine (mg/dL)				
postoperative	0.83 ± 0.35	*p = .440*	0.77 ± 0.25	*p = .493*
preoperative	0.81 ± 0.35		0.80 ± 0.25	
Serum cystatin C (mg/L)				
preoperative	1.18 ± 0.39	*p* = .581	1.13 ± 0.30	*p* = .306
postoperative	1.20 ± 0.40		1.17 ± 0.32	
eGFR (ml/min/1.73m^2^)				
preoperative	82.1 ± 26.1	*p* = .161	82.1 ± 23.0	*p* = .581
postoperative	78.6 ± 22.9		80.2 ± 21.5	
uACR (mg/mmol)				
preoperative	22.9 ± 39.8	*p* = .002	22.6 ± 47.3	*p* < .001
postoperative	13.0 ± 23.5		8.3 ± 28.5	
sUMOD (ng/ml)				
preoperative	76.02 ± 35.80	*p* = .025	91.77 ± 45.35	*p* = .819
postoperative	85.56 ± 40.74		93.12 ± 41.58	
sKIM-1 (pg/ml)				
preoperative	121.05 ± 134.69	*p* = .028	104.39 ± 95.96	*p* = .475
postoperative	84.40 ± 120.68		107.50 ± 215.04	
HbA1c (%)				
preoperative	6.9 ± 1.6	*p* < .001	7.3 ± 2.6	*p* < .001
postoperative	5.9 ± 1.1		5.8 ± 1.3	
RR systolic (mmHg)				
preoperative	147.8 ± 16.7	*p* = .021	148.6 ± 20.3	*p* < .001
Postoperative	138.4 ± 14.4		132.9 ± 19.2	
RR diastolic (mmHg)				
preoperative	89.3 ± 12.8	*p* = .136	90.6 ± 12.2	*p* = .059
postoperative	85.2 ± 10.1		83.7 ± 11.9	
BMI (kg/m^2^)				
preoperative	47.2 ± 5.3	*p* < .001	47.1 ± 6.8	*p* < .001
Postoperative	36.4 ± 6.6		32.2 ± 5.5	
%TWL (%)	23.6 ± 9.4		31.0 ± 11.6	*P* = .032

*SG *Sleeve gastrectomy, *RYGB* Roux-en-y gastric bypass, *eGFR* estimated glomerular filtration rate, *uACR* urinary albumin-creatinine ratio, *sUMOD* serum uromodulin, *sKIM-1* serum kidney injury molecule-1, *HbA1c* Glycated hemoglobin,* RR* Blood pressure, *BMI* Body mass index, *%TWL* Percentage total weight loss

### Exploratory Analysis by Nephropathy Subtype

Analyses by nephropathy subtype were exploratory in nature due to the small number of patients within individual subgroups.

**Fig. 4:**
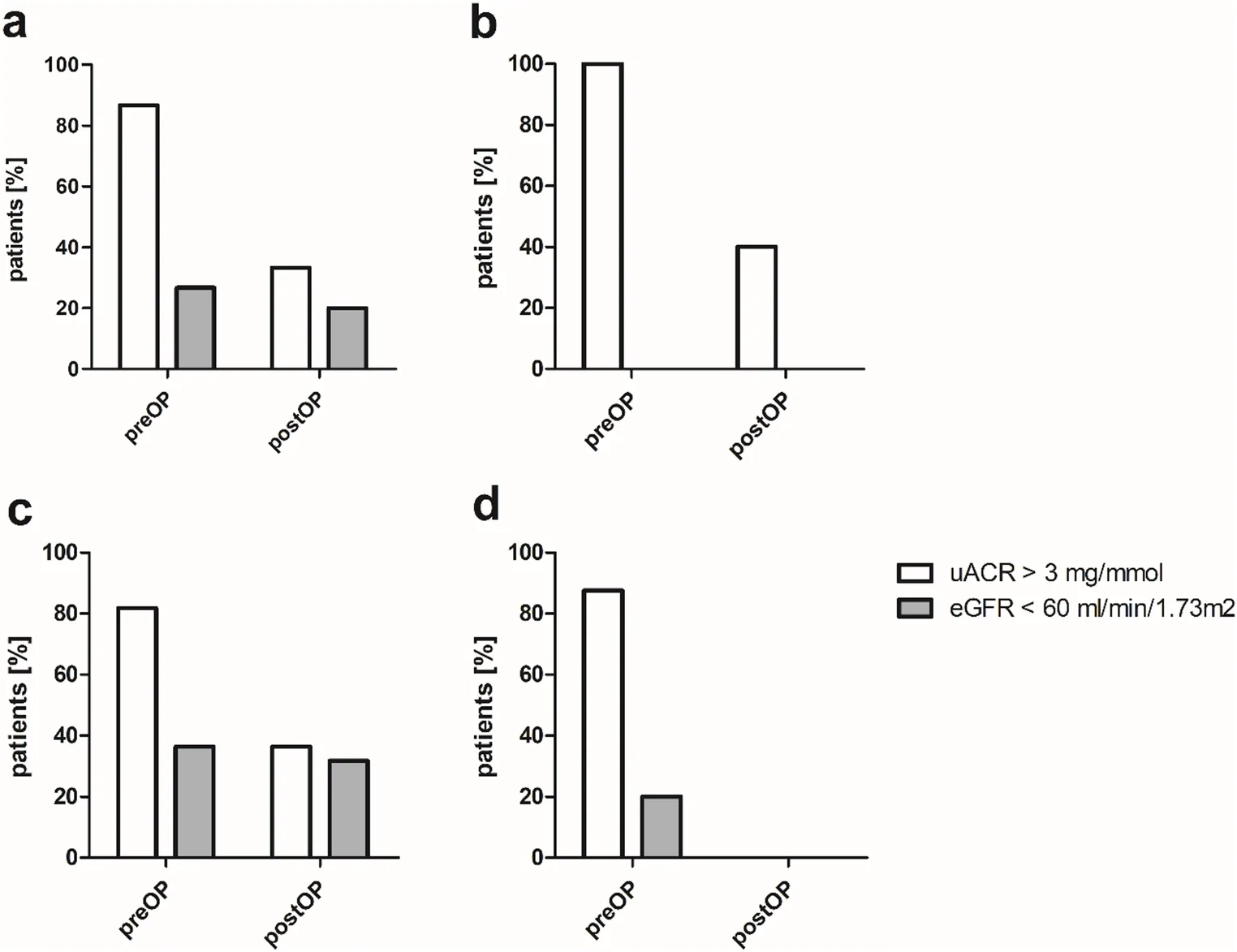
Exploratory pre- versus postoperative development of microalbuminuria (urinary albumin-creatinine ratio > 3 mg/mmol) and reduced eGFR (estimated glomerular filtration rate < 60 ml/min/1.73m^2^) in subgroups preoperatively presenting only arterial hypertension (**a**), only type 2 diabetes mellitus (**b**), both (**c**) and none (**d**) of the conditions. White bars indicate albuminuria and shaded bars indicate reduced eGFR as defined above

With respect to hypertensive, diabetic and obesity-related subtypes of nephropathy, 16% of patients had neither hypertension nor T2D (SG 12% vs. RYGB 20%) at baseline. Additionally, 30% had hypertension alone (SG 36% vs. RYGB 24%), 20% had isolated T2D (SG 8% vs. RYGB 12%) and 44% presented with both conditions (SG 44% vs. RYGB 44%). Patients with neither hypertension nor T2D were significantly younger than patients with one or both conditions (40.1 ± 7.2 vs. 49.5 ± 8.7 years, *p* = .011). Preoperative BMI was similar across all etiological subgroups (*p* = .427). Resolution of renal involvement occurred more frequently in patients without preoperative T2D compared to those with T2D (73.9% vs. 44.4%, *p* = .035). Likewise, resolution was more common in patients without preoperative arterial hypertension than in those with hypertension (84.6% vs. 48.6%, *p* = .024). All patients without hypertension or T2D (SG *n* = 3, RYGB *n* = 5), who likely had obesity-related glomerulopathy (ORG), experienced resolution of renal involvement. Patients without preoperative hypertension or T2D exhibited greater increasev in sUMOD (+ 14.51 ± 18.79 vs. +1.44 ± 20.51 ng/ml, *p* = .039), higher postoperative eGFR (85.8 ± 16.2 vs. 72.1 ± 25.2 ml/min/1.73m^2^, *p* = .049), and a significantly higher rate of nephropathy resolution (100% vs. 41%, *p* = .040) compared to those with both hypertension and T2D. All etiological subgroups demonstrated comparable benefit from SG relative to RYGB. However, subgroup sizes were small and findings should be interpreted cautiously. The evolution of albuminuria and aGFR across nephropathy subgroups is shown in Fig. 4.

### Potential Predictors for Nephropathy Resolution

Among patients undergoing RYGB, those who achieved renal recovery had a significantly lower preoperative age (45.2 ± 7.9 vs. 54.0 ± 8.4 years, *p* = .031), greater %TWL (34.0 ± 12.2 vs. 24.6 ± 7.3%, *p* = .037), and a larger decrease in systolic blood pressure (-21.9 ± 15.9 vs. -5.1 ± 14.3 mmHg, *p* = .020) compared to those with persistent nephropathy. Preoperative sUMOD levels were significantly higher in patients with full resolution of renal involvement after both SG (97.3 ± 33.8 vs. 56.4 ± 25.4 ng/ml, *p* = .002) and RYGB (103.3 ± 43.6 vs. 67.3 ± 41.2 ng/ml, *p* = .037). In exploratory logistic regression analysis, higher preoperative sUMOD levels were associated with postoperative resolution of renal involvement (*p* = .018; Table 3).

**Table 3:** Exploratory Logistic Regression Analysis for Postoperative Resolution of Renal Involvement

	b	SE	Wald X^2^	*P* <	OR	95% CI	
Age	0.076	0.054	1.928	0.165	1.079	0.969	1.200
Δ BMI	0.177	0.093	3.587	0.058	1.193	0.994	1.433
Δ RRsys	− 0.018	0.024	0.587	0.444	0.966	0.937	1.029
sUMOD preop	− 0.034	0.014	5.595	0.018	1.001	0.940	0.994
sKIM-1 preop	0.001	0.003	0.089	0.766	1.658	0.994	1.008

Note: Cox & Snell R^2^ = 0.382, Nagelkerkes R^2^ = 0.515

*b *regression coefficient, *Wald X*^*2 *^Wald chi square test, *SE *standard error,* OR* odds ratio, *95% CI* 95% confidence interval, *Δ BMI * Reduction of body mass index, *Δ RRsys* Reduction of systolic arterial blood pressure, *sUMOD preop *preoperative serum uromodulin levels, *sKIM-1 preop* preoperative serum kidney injury molecule-1 levels

## Discussion

This study demonstrates that both SG and RYGB significantly improve nephropathy, albuminuria, glycemic control, and blood pressure in patients with obesity and kidney impairment, with no difference between procedures. While uACR was the marker with the clearest postoperative improvement after MBS, it also represents a clinically meaningful and well-established surrogate of renal damage and disease progression, while traditional markers like serum creatinine and eGFR remained stable, highlighting their limitations in this population. Notably, novel biomarkers – sUMOD and sKIM-1 – improved only after SG, suggesting potential differences in renal recovery mechanisms. Patients without hypertension or T2D, likely with obesity-related glomerulopathy, had higher nephropathy resolution rates and greater sUMOD increases. Preoperative sUMOD was also a significant predictor of renal improvement. These findings align with previous studies indicating that traditional renal markers and eGFR formulas have limited diagnostic accuracy in patients with obesity [21, 22]. Friedman et al. suggested the CKD-EPI combined creatinine-cystatin C formula as a more accurate tool for estimating GFR in patients with obesity, yet its values can still deviate by up to 30% from true GFR [21]. Moreover, factors like protein intake and blood pressure further complicate eGFR assessment after weight loss [23, 24]. This highlights the need for more reliable tools to evaluate renal function in obesity. Biomarkers such as sUMOD and sKIM-1, which directly reflect nephron mass and function, may offer better insights. Unlike traditional markers, they are less influenced by obesity-related glomerular hyperfiltration, which often leads to falsely elevated eGFR despite ongoing kidney damage [25]. The importance of albuminuria as a predictor for progressive kidney damage is well established. In ORG, subnephrotic proteinuria often appears early and reflects obesity-induced renal injury [25]. Elevated proteinuria is independently associated with increased risk of CKD progression to renal failure, whereas the relationship between low GFR and outcomes is less consistent [26, 27]. Therefore, our finding that MBS significantly reduces albuminuria is highly relevant. As albuminuria is a common marker across various forms of nephropathy, its resolution suggests a broader renoprotective effect of surgery, beyond improvements in eGFR alone. In this study, subgroup analyses were limited to patients with presumed obesity-related glomerulopathy, hypertensive nephropathy, or diabetic nephropathy, and no cases of biopsy-proven glomerulonephritis or other renal diseases were included. Patients likely affected by ORG showed the highest rates of nephropathy resolution. Although ORG is a histological diagnosis, it may be clinically inferred in younger patients with obesity who lack comorbid conditions such as hypertension and T2D. Our findings suggest that these patients may represent a subgroup particularly responsive to MBS. Therefore, given their high rates of nephropathy remission, early surgical intervention should be considered in these individuals to prevent progression to more advanced kidney damage. However, longer follow-up is needed to assess the durability of MBS effects, especially in younger patients. While our study found higher remission of albuminuria than eGFR recovery, it is unclear whether these improvements are sustained long-term. In a study by Cohen et al., RYGB showed superior albuminuria control after 2 years compared to medical treatment in patients with T2D and a BMI of 30–35 kg/m^2^, but this effect diminished after 5 years [13, 14]. Although our sample size limits conclusions on end-stage kidney disease, large-scale data from the Swedish Obese Subjects Study demonstrated that MBS significantly reduces the incidence of diabetic end-stage kidney failure compared to medical therapy alone [28]. Additionally, MBS also achieved significant improvements in key drivers of CKD (albuminuria, HbA1c and blood pressure) in subgroups with hypertension and T2D [29, 30]. This was reflected in the impressive reduction or discontinuation of antihypertensive and antidiabetic medication at 2.5 years follow-up. These changes likely contribute to improvements in uACR that appear independent of weight loss, consistent with previous reports [31]. Among novel markers of kidney function, sUMOD improved significantly after SG and was consistently higher in patients with nephropathy resolution after both procedures. Furthermore, higher preoperative sUMOD levels were consistently associated with renal recovery and may represent a promising candidate biomarker, warranting further investigation in larger studies. In contrast, baseline sKIM-1 levels were elevated across all cohorts compared to healthy individuals described by Sabbisetti et al. with mean levels of 64.4 (51–77.7, 95% CI) [18], though normative data remain limited. Notably, sKIM-1 significantly decreased only after SG in this study, for reasons that remain unclear. The different response between SG and RYGB in novel markers should be confirmed in larger cohorts. A key strength of this study lies in its direct comparison of SG and RYGB in patients with preoperative nephropathy, using both traditional and novel biomarkers, with a robust 2.5-year follow-up.

Several limitations should be acknowledged, including relatively small sample size, lack of kidney biopsy data, and no information of the duration of comorbidities such as T2D and hypertension. First, the overall sample size of this study is limited, which restricts statistical power and the generalizability of our findings. Likewise, subgroup analyses according to nephropathy etiology were limited by the small number of patients within individual groups and should therefore be regarded as exploratory and descriptive. Consequently, no definitive conclusions regarding differential effects between nephropathy subtypes can be drawn, and these observations require confirmation in larger, adequately powered cohorts. Similarly, the exploratory logistic regression analysis must be interpreted with caution given the limited sample size, which increases the risk of overfitting and imprecision of effect estimates. Accordingly, regression findings are intended to be hypothesis-generating rather than confirmatory and warrant validation in larger studies.

Second, the lack of kidney biopsy data and the lack of information of the duration of relevant comorbidities, including T2D and hypertension, are limitations to our results. Nonetheless, prior research has shown that even patients with long-lasting insulin-dependent T2D may experience improvement or reversal of kidney impairment after MBS [11]. Even though propensity score matching balanced baseline characteristics between SG and RYGB groups, patients were not matched by nephropathy subtype, which may influence response to surgery. However, subgroup analyses showed comparable benefits across subtypes, mitigating this limitation. Future studies with larger samples should consider matching or stratifying by nephropathy subtype.

Furthermore, albuminuria was assessed using a single uACR measurement at each time point. While uACR is a well-established marker of renal injury, it is subject to biological variability, and clinical diagnosis of persistent albuminuria requires confirmation on repeated measurements. Therefore, uACR-based outcomes in this study are reported as microalbuminuria remission rather than definitive nephropathy. Nevertheless, the consistent and significant reduction in uACR observed across both surgical groups, together with concordant improvements in blood pressure, glycemic control, and novel renal biomarkers, supports a true improvement in renal status rather than random variation.

Based on our findings, we advocate for timely consideration of MBS in patients with refractory obesity and impaired kidney function, given its consistent renoprotective effects and potential to delay or prevent progression to end-stage renal disease (ESRD) [11, 32]. Younger patients likely affected by ORG appear to benefit particularly, regardless of type of surgery. These findings support a shift in clinical thinking - impaired kidney function should not preclude MBS, but rather strengthen its indication to prevent progression to more severe stages of kidney damage early. Confirmation in larger, long-term studies is needed.

## Conclusion

This study suggests that MBS effectively improves renal involvement, including remission of microalbuminuria, in patients with obesity, regardless of the surgical procedure. Patients likely affected by obesity-related glomerulopathy appear especially responsive, highlighting the potential value of early surgical intervention. In patients with T2D and hypertension, improvements in albuminuria, HbA1c, and blood pressure suggest benefits beyond weight loss. SUMOD emerges as a promising renal marker for assessing kidney function and predicting recovery. These results support MBS as a therapeutic option for nephropathy in obesity. Larger, long-term trials are needed to confirm these findings and evaluate the impact on CKD progression and ESRD risk.

## Supplementary Information

Below is the link to the electronic supplementary material.

Supplementary Material 1

## Data Availability

The data that support the findings of this study are available from the corresponding author, but restrictions apply to the availability of these data, which were used under licence for the current study and so are not publicly available. The data are, however, available upon request and with the permission of the corresponding author.
