# Supplementary material for: Renal Outcomes After Sleeve Gastrectomy versus Gastric Bypass in Patients with Nephropathy: Results from a Retrospective Study
Source: Obes Surg. 2026 Apr 21;36(6):2947–56. doi: 10.1007/s11695-026-08684-4 (PMC13653830; doi:10.1007/s11695-026-08684-4)
Supplement: Supplementary file 1 [file 11695_2026_8684_MOESM1_ESM.docx]

**Supplementary Materials**

*Methods*

Surgical treatment was indicated according to the currently accepted criteria for metabolic bariatric surgery (MBS) after a multidisciplinary work up and interdisciplinary indication for MBS in a metabolic board. Both procedures were standardized and performed laparoscopically by the same surgical team. A RYGB with a 75cm biliopancreatic and a 150cm alimentary limb was performed. Mesenteric defects were closed in all patients routinely. SG was performed using a 42 French bougie starting 6cm proximal of the pylorus. The proximal staple line was oversutured in all cases. All patients undergoing surgery received a full endocrinological work-up preoperatively to rule out organic causes for obesity. Antihypertensive, antidiabetic medication and insuline treatment were suspended immediately after surgery and, if needed, resumed according to the recommendation of internal medicine specialists. Blood pressure and serum glucose were regularly monitored. Antidiabetic medication in all cohorts was conducted according to the currently valid national S3 guideline for treatment of type 2 diabetes. Blood samples were obtained preoperatively and after a follow up period of approximately 2.5 years. Serum samples for analysis of sUMOD and sKIM-1 were stored at -80°C immediately after blood extraction before measurements were performed. Customary enzyme-linked immunosorbent assay (ELISA) kits based on monoclonal antibodies were used for quantification of both marker proteins, following the manufacturer’s instructions (Invitrogen, ThermoFisher Scientific, Waltham, MA, USA). Two measurements were taken of all ELISA samples to further analyse their mean value. A microplate reader (Infinite 200 Pro, Tecan, Mainz, Germany) measured absorption at 450nm and Magellan data analysis software (Tecan, Mainz, Germany) processed ELISA results. All other parameters were measured at the certified central laboratory of the University Hospital using serum specimens and spontaneous urine samples as part of the routine laboratory workup.
